# Could low birth weight and preterm birth be associated with significant burden of hip osteoarthritis? A systematic review

**DOI:** 10.1186/s13075-018-1627-7

**Published:** 2018-06-08

**Authors:** Sultana Monira Hussain, Ilana N. Ackerman, Yuanyuan Wang, Ella Zomer, Flavia M. Cicuttini

**Affiliations:** School of Public Health and Preventive Medicine, Monash University, Melbourne, VIC 3004 Australia

**Keywords:** Low birth weight, Preterm birth, Hip osteoarthritis, Total hip arthroplasty, Economic evaluation

## Abstract

**Background:**

Approaches for the prevention and treatment of hip osteoarthritis (OA) remain limited. There are recent data suggesting that low birth weight (LBW) and preterm birth may be risk factors for hip osteoarthritis. This has the potential to change the current paradigm of hip osteoarthritis prevention by targeting early life factors. The aim of this review was to examine the available evidence for an association of LBW and preterm birth with hip OA. The potential cost implications associated with total hip arthroplasty were also evaluated.

**Methods:**

Ovid Medline, EMBASE, and Cinahl were searched up until August 2017 using MeSH terms and key words. Methodological quality was evaluated using the National Heart Lung and Blood Institute (NHLBI) quality assessment tool. Qualitative evidence synthesis was performed to summarise the results. Bradford Hill’s criteria for causation including the temporal relationship, consistency, strength of the association, specificity, dose-response relationship, and analogy were used to assess the evidence for causation. Economic modelling was used to calculate the potential economic burden associated with LBW or preterm birth related total hip arthroplasty using Australian data from 2012 to 2015.

**Results:**

Five studies, ranging from high to low quality, were included. Hip bone shape abnormalities examined included developmental hip dysplasia and immature hip, and hip osteoarthritis included osteophytes and total hip arthroplasty. A causal link between low birth weight or preterm birth and hip osteoarthritis was found. Of the 30,477 total hip arthroplasties performed for hip osteoarthritis in Australia in 2015, 5791 were estimated to be born preterm and 5273 with low birth weight. This equated to a potential total hip arthroplasty cost of AU$145,136,082 and AU$132,150,222 for these subgroups, respectively.

**Conclusion:**

Available data suggest that low birth weight and preterm birth are associated with hip bone shape abnormalities and hip osteoarthritis requiring total hip arthroplasty, with a substantial associated financial burden. Given the current lack of effective treatment and prevention strategies for hip osteoarthritis, this offers a new avenue for reducing the future burden of hip osteoarthritis.

**Electronic supplementary material:**

The online version of this article (10.1186/s13075-018-1627-7) contains supplementary material, which is available to authorized users.

## Background

Hip osteoarthritis (OA) is a common joint disease with one in four people developing symptomatic hip OA in their lifetime [1]. There are limited treatment and preventive strategies for hip OA; as a result, end-stage disease is treated by total hip arthroplasty (THA), imposing a burden on health systems internationally [2, 3]. In Australia, almost one in eight people have a lifetime risk of undergoing THA [3]. The number of THAs per annum in Australia has risen steadily over time, with an increase of 65% from 2003 to 2015 [4]. To reduce the burden of this disease, new approaches to prevention are needed.

There is increasing evidence for the importance of hip bone shape in the pathogenesis of hip OA [5, 6]. For example, recent studies have shown that mild acetabular dysplasia [7] and alterations in hip bone shape and geometry [6] predate the onset of hip OA. Events in early life may be risk factors for hip OA and these may be mediated by changes in hip bones. Potential mechanisms affecting the bone include differences in bone accretion for a fetus in an intrauterine environment versus a preterm infant in an extrauterine environment [8], and greater bone mineral content in a full-term-born baby compared with a preterm infant [9, 10]. Both low birth weight (LBW) and preterm born babies frequently suffer from metabolic bone disease which is often asymptomatic and self-limiting [11] and has been linked to thickening of the chondrocostal junctions of the long bones [12]. Preterm birth has also been linked to reduced bone mass [13] and underdeveloped acetabula [14]. Post-delivery, these infants demonstrate an altered hip position (with hip extension) compared with the position of intrauterine life (flexed and abducted hip) and this may contribute to an increased incidence or greater severity of acetabular dysplasia [15, 16]. It is perhaps unsurprising, therefore, that LBW and preterm birth have been associated with the pathology of hip OA. If this association exists between LBW, preterm birth, and hip structure, it could facilitate a paradigm shift for the monitoring and prevention of hip OA. Hip joint abnormalities exist as a continuum, and subtle morphological abnormalities of hip bone, i.e. mild acetabular dysplasia or a shallow acetabulum, are associated with late-onset of primary hip OA [17, 18]. As hip OA is a chronic long-term condition, to understand the risk factors and pathogenesis of hip OA we need to consider the stages of hip OA across the life-course from early changes in hip bone shape to established hip OA and end-stage joint disease requiring joint replacement surgery. Without considering these earlier stages, simply trying to identify hip OA will not be helpful as the prevalence of hip OA in those aged < 40 years is very low.

A substantial number of babies are born with LBW in developed countries. Based on data from 2015 and 2016, 6.2% of infants are born LBW (< 2500 g) and 8% are born preterm (at < 37 completed weeks gestation) in Australia [19], which is lower than the prevalence of LBW and preterm births in the United States (8.0% LBW and < 12.0% preterm) [20, 21], the United Kingdom (7.6% LBW) [22], and the Organisation for Economic Co-operation and Development average (6.6% LBW) [23]. A steady increase in the number of LBW and preterm born infants is expected since the average age of mothers in developed countries is increasing [24]; older mothers (≥ 35 years) [25] are at higher risk of delivering LBW and preterm born babies than mothers aged 20 to 34 years. Additionally, the survival rate of LBW and preterm birth infants has increased almost 80% in the past 30 years [9]. Thus, determining whether LBW or preterm birth increases the risk of abnormal hip bone shape and hip OA, and subsequent THA in later life and the potential economic burden, will be important to inform future resource allocation and hip OA prevention initiatives.

In this study, we aimed to examine the available evidence for an association of LBW and preterm birth with hip OA, and to examine the evidence for potential causation based on the Bradford Hill criteria. Considering the range of definitions used to describe OA-related changes and outcomes, we have collectively termed ‘hip pathologies’, hip joint abnormalities, hip OA, and hip arthroplasty for hip OA as ‘Hip OA’. Given the huge burden associated with both hip OA and LBW and preterm birth, we also considered the potential cost implications associated with THA.

## Methods

### Systematic review

This systematic review was conducted and reported in accordance with the Preferred Reporting Items for Systematic Review and Meta-Analysis (PRISMA) guidelines [26].

#### Search strategy

Ovid Medline, CINAHL, and EMBASE databases were searched between January 1947 and August 2017 using MeSH terms (after exploding) and key words to identify studies examining the association between LBW or preterm birth and hip bone abnormality and hip OA. The MeSH and key terms used to define hip bone abnormality and hip OA included “hip” and “osteoarthritis” or “degenerative arthritis” or “coxarthritis” or “dysplasia” or “joint space narrowing” or “osteophytes” or “bone shape” or “bone geometry” or “neck shaft angle” or “hip deformity” or “femora acetabular impingement” or “pincer deformity” or “cam deformity”. For LBW and preterm birth, the MeSH and key terms used were “birth weight” or “low birth weight” or “preterm birth” or “prematurity” or “preterm”. Searches were limited to human studies and those published in English.

#### Study selection

Two authors (SMH and YW) independently assessed study eligibility using a three-stage determination method, reviewing the title, abstract, and then full text. Any disagreement between the two authors was resolved by consensus reviewing the criteria. Studies were only included if they assessed hip bone abnormality and hip OA including developmental dysplasia of the hip (DDH), hip instability at childhood, hip deformity, hip osteophytes, joint space narrowing, symptomatic hip OA, radiological hip OA, magnetic resonance imaging (MRI) changes of the hip, hip bone shape, hip bone geometry, hip neck-shaft angle, hip deformity, THA for hip OA, and either LBW or preterm birth or both LBW and preterm birth. Case reports, conference abstracts, and review articles were excluded, as were studies without a comparison group. The reference lists of the included articles and review articles identified were searched to identify any additional relevant studies.

#### Data extraction and synthesis

Two authors (SMH and YW) extracted data independently on study design, participant characteristics (number, gender, and age), definition and prevalence of hip bone abnormality and hip OA, duration of follow-up, measures of LBW or preterm birth, adjustment for confounding factors, and associations between LBW or preterm birth and hip bone abnormality and hip OA. A third author (FMC) assessed the consistency of extracted data. Although a meta-analysis was planned, significant heterogeneity across the studies (predominantly different ages of the study populations, different methods for defining and assessing hip bone abnormality and hip OA, and different sources of birth weight data) precluded the pooling of data for analysis.

#### Risk of bias assessment

Two authors (INA and YW) independently assessed the risk of bias of included studies using the National Heart Lung and Blood Institute (NHLBI) quality assessment tool for observational studies [27]. This tool includes 14 criteria for cohort and cross-sectional studies and 12 criteria for case-control studies to assess the internal validity and risk of bias, and scores the quality of a study as ‘high’ (low risk of bias), ‘fair’ (moderate risk of bias), or ‘low’ (high risk of bias).

#### Bradford Hill criteria for causation

The Bradford Hill criteria [28] were used to examine the evidence for a causal relationship between LBW and hip OA, and between preterm birth and hip OA. These criteria are commonly used to assess the adequacy of evidence for a causal relationship between an exposure and a consequence. These criteria include temporal relationship, consistency, strength of the association, specificity, dose-response relationship, and analogy.

### Economic evaluation

An economic evaluation was undertaken to estimate the costs associated with THA likely attributable to LBW or preterm birth at a national level. Trends in the national prevalence of LBW and preterm birth were examined using annual birth data published by the Australian Institute of Health and Welfare [29, 30]. Data from Australian Orthopaedic Association National Joint Replacement Registry annual reports were used to establish the incidence of THA performed for OA in 2015 [31]. Data on the likelihood of having THA due to LBW or preterm birth were obtained from a recent Australian data linkage study [32], with additional summary data provided by the authors. These proportions were applied in separate calculations to annual THA incidence data to estimate the number of THA procedures performed for the LBW and preterm birth populations in 2015. The costs associated with THA were estimated from a health system perspective [33]. Published episode of care costs for THA were inflated to 2016 prices (AUD $22,817 per THA) [33] and multiplied by the number of estimated THA procedures in 2015 to calculate the total cost of THA.

## Results

### Systematic review

#### Search results

**Fig. 1:**
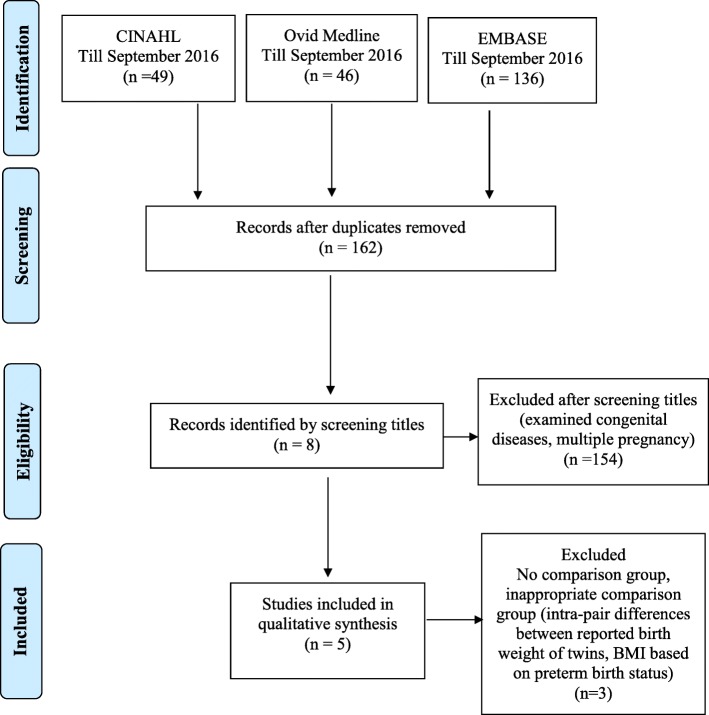
PRISMA flow diagram of included articles. BMI body mass index

Database searches identified 231 records (CINAHL, *n* = 49; Ovid Medline, *n* = 46; and EMBASE, *n* = 136), of which 69 articles were duplicates. Of the remaining 162 articles, 154 were excluded after title and abstract screening as these studies did not assess hip OA in relation to LBW or preterm birth. Full-text screening was performed for eight articles. Three were excluded as they did not include an appropriate comparison, leaving five included studies (Fig. 1). No additional articles were identified from reference lists.

#### Summary of included studies

Table 1 provides an overview of the included studies. The included studies comprised two cohort [32, 34], one case-control [35], and two cross-sectional studies [36, 37] published between 1993 and 2015. Two studies originated from Australia [32, 35], two from the UK [34, 36], and one from Turkey [37]. All the studies included men and women.

**Table 1:** Characteristics of studies included in the systematic review

Author, country, and year	Study population and % women	Age of study population (mean ± SD)	Hip OA or hip pathologies related to hip OA	Prevalence % outcome (OA, DDH, α-angle, hip deformity) (men, women)	Follow-up time (mean ± SD)	Quality of study^a^
Hip bone shape abnormality						
Case-control study						
Chan et al., Australia, 1997 [35]	All live births during 1986–93 *n* = 151,257 47%	28 days to 5 years	Self-reported DDH, birth registry	0.75% DDH	N/A	Low
Cross-sectional studies						
Orak et al., Turkey, 2015 [37]	Infants born in one hospital *n* = 467 44%	Preterm 31.11 ± 2.51 weeks Term 40.22 ± 0.36 weeks	α-angle < 60 ° of the hip joint suggestive of immature or pathologic hip	NR	N/A	Low
Davis et al., UK, 1993 [36]	Infants born in one hospital *n* = 33 55%	3–4.5 years	Hip deformity by footprint angle and hip rotation	NR	N/A	Low
Hip OA						
Cohort studies						
Hussain et al., Australia 2015 [32]	*n* = 3604 participants 60%	No arthroplasty 51.8 ± 10.0 Hip arthroplasty 59.0 ± 9.5	Hip arthroplasty for hip OA	2.1%	9.3 ± 2.1	High
Clynes et al., UK 2014 [34]	*n* = 444 50%	Median 75 years (IQR 73–77)	American College of Rheumatology algorithm to define hip OA Radiographic Kellgren and Lawrence (KL) score of hip to count osteophytes	Men 3.2% Women 6.0%	13 years	Fair

*DDH* developmental dysplasia of the hip, *IQR* interquartile range, *N/A* not applicable, *NR* not reported, *OA* osteoarthritis, *SD* standard deviation

^a^ Evaluated using the National Heart Lung and Blood Institute (NHLBI) quality assessment tool for observational studies [27]

#### Assessment of hip pathology and hip OA

Hip bone abnormality included self-reported DDH [35], α-angle of the hip joint (suggestive of immature or pathologic hip) by ultrasound examination [37], and hip deformity by footprint angle and hip rotation [36]. Hip OA was defined by American College of Rheumatology (ACR) algorithms based on the presence of osteophytes and overall Kellgren and Lawrence (K-L) score assessed from hip radiographs [34], and hip arthroplasty for OA [32].

#### Assessment of low birth weight or preterm birth

Two studies reported both LBW and preterm birth data [32, 36], while the remainder collected data only on LBW [34, 35] or preterm birth [37]. Birth weight information was collected from hospital records [36, 37] and birth registries [34, 35] for four of the included studies. One study collected self-reported birth weight data and whether the participant was born ≥ 2 weeks preterm [32]. In this study, 10% of the participants had birth weight data from hospital records compared against their self-reported data for validation [32].

#### Prevalence of hip bone abnormality and hip OA

One study reported that self-reported DDH was present among 0.75% of the population [35]. Two studies [32, 34] reporting hip OA prevalence showed a 2.1% prevalence when hip OA was defined by THA [32], and 3.2% for men and 6.0% for women when the disease was defined as clinical hip OA [34].

#### Risk of bias

A risk of bias assessment was undertaken for each of the included studies. The overall quality assessment is shown in Table 1, with details of quality assessment presented in Additional file 1: Table S1 for cohort and cross-sectional studies and Additional file 1: Table S2 for case-control studies. Of the two cohort studies, one was classified as high quality [32] and the other was classified as fair quality [34]. The other studies, one case-control [35] and two cross-sectional [36, 37] studies, were considered of low quality. Limitations associated with the cohort studies included the categorisation of birth weight data (i.e. birth weight data was not treated as a continuous variable) [32] and the lack of sample size justification and loss to follow-up [34]. The two cross-sectional studies did not provide information on temporality and also failed to adjust for potential confounders [36, 37]. In the case-control study, the processes used to identify or select cases and controls was problematic, and the process for measuring exposure was not clearly defined [35]. The study population and research question were defined clearly in all the studies.

#### Association between LBW or preterm birth and hip bone abnormality

One low-quality case-control study found that participants born with LBW were less likely to develop DDH [35]. The two low-quality cross-sectional studies reported conflicting results regarding birth weight and hip bone abnormality. One study showed that LBW and preterm birth were associated with deformation of the lower limb including the hip [36], while the other study found that preterm babies had less prevalence of abnormal α-angle of the hip joint (< 60 °, suggestive of immature hip development) compared with full-term babies [37]. Neither study adjusted for potential confounders (Table 2).

**Table 2:** Association between low birth weight or preterm birth and hip pathology/osteoarthritis

Author and year	Low birth weight/preterm measurement	Confounder adjusted for	Results	Conclusion
Hip bone shape abnormality				
Case-control study				
Chan et al., 1997 [35]	Birth weight from the birth registry	Maternal age, region of residence, parity, oligohydramnios, presentation and method of delivery, baby’s sex, birth weight, gestation	Low birth weight and DDH, where birth weight 3000–3500 g is referent Birth weight < 2000 g OR 0.30 (95% CI 0.12–0.77) Birth weight 2000–2500 g OR 0.52 (95% CI 0.31–0.88)	Those who were born with low birth weight (< 2500 g) were less likely to develop DDH
Cross-sectional studies				
Orak et al., 2015 [37]	Hospital-recorded birth weight	Unadjusted	Preterm born and α-angle of the hip joint suggestive of immature or pathologic hip Preterm born babies with α-angle < 60 ° = 2.7% Full-term born babies with α-angle < 60 ° = 28.5% (*p* < 0.001, Fisher’s exact test)	These results suggest that prematurity is not a predisposing factor for immature hip predictive of DDH
Davis et al., 1993 [36]	Hospital-recorded birth weight	Unadjusted	Low birth weight and preterm birth with hip deformity Out-toeing 62% in low birth weight vs 35% in the term babies Total rotation of hip preterm group 119.20 (19.6) vs term group 99.20 (9.6) (*p* < 0.003)	Deformation of the lower limb including hip frequently seen in preterm babies during early infancy
Hip OA				
Cohort studies				
Hussain et al., 2015 [32]	Self-reported birth weight and whether born ≥ 2 weeks preterm	Age, sex, BMI, hypertension, diabetes mellitus, smoking, and physical activity	Low birth weight and hip arthroplasty HR 2.02 (95% CI 1.10–3.73) Preterm birth HR 2.53 (95% CI 1.30–4.92)	Individuals born with LBW or at preterm are at increased risk of hip arthroplasty for OA in adult life
Clynes et al., 2014 [34]	Birth weight from the birth registry	Age, sex, BMI, smoking and alcohol	Lower birth weight and radiographic hip OA OR 0.78 (95% CI 0.48–1.27) Lower birth weight andosteophytes in hip OR 1.51 (95% CI 1.13–2.01)	Individuals with lower birth weights were more likely to have hip osteophytes but not hip arthritis

*BMI* body mass index, *CI* confidence interval, *DDH* developmental dysplasia of the hip, *HR* hazard ratio, *LBW* low birth weight, *OA* osteoarthritis, *OR* odds ratio

#### Association between LBW or preterm birth and hip OA

Two cohort studies examined the relationship between LBW or preterm birth and hip OA [32, 34] (Table 2). In a high-quality cohort study, LBW and preterm birth were independently associated with increased incidence of THA for OA [32]. A stronger association was evident for preterm birth than for LBW [32]. The other fair-quality cohort study showed that lower birth weight (as a continuous variable) was associated with hip osteophytes but not clinical hip OA (defined using algorithms developed by the ACR) [34].

#### Evidence for causation using the Bradford Hill criteria for causation

Table 3 presents the Bradford Hill criteria, an explanation of each criterion, and evidence for a causal relationship between LBW, preterm birth, and hip OA in relation to each criterion. While there was evidence for causation with respect to six items of the Bradford Hill criteria (consistency, strength of the association, dose-response relationship, specificity, analogy, and temporal relationship), there was no or limited evidence for the remaining three criteria (plausibility, reversibility, and coherence). Overall, this approach suggests that there is modest evidence for a cause-effect relationship, with most of the criteria being present between LBW, preterm birth, and hip pathology/hip OA.

**Table 3:** Evidence for a causal relationship between low birth weight and preterm birth and hip osteoarthritis according to the Bradford Hill criteria

Bradford Hill criterion and description	Hip osteoarthritis
Temporal relationship This is an essential criterion. For a possible risk factor to be the cause of a disease, it must come before the disease. This is generally easier to establish from cohort studies than from cross-sectional or case-control studies, when measurements of the possible cause and the effect are made at the same time	Criterion met: Yes Hussain et al. [32] In a cohort study people born with low birth weight (LBW) or preterm underwent hip arthroplasty for hip osteoarthritis (OA) at an average age of 59.0 (standard deviation (SD) 9.5) years Clynes et al. [34] Participants of the Hertfordshire Cohort Study who were born LBW had more osteophytes in the hip joint detected by x-ray at the age median of 75 (interquartile range (IQR) 73–77) years
Plausibility A risk factor associated with a disease is more likely to be the cause of the disease if the association found is consistent with knowledge obtained from other sources, such as animal experiments and experiments on biological mechanisms. However, this criterion must be used with care as a lack of plausibility may simply reflect a lack of scientific knowledge	Criterion met: No
Consistency If similar results have been found in different populations using different study designs, the association is more likely to be causal as it is unlikely that all studies were subject to the same types of errors (chance, bias or confounding). However, a lack of consistency does not exclude a causal association, as different exposure levels and other conditions may reduce the impact of the causal factor in certain studies	Criterion met: Yes Different stages of hip OA including hip arthroplasty for OA [32], osteophytes in hip joint [34], and hip shape deformity [36] were found in different populations using different study designs, including two cohort studies [32, 34] and a cross-sectional study [57]
Strength of an association The strength of an association is measured by the size of the relative risk. A strong association is more likely than a weak association to be causal, as a weak association could more easily be the result of confounding or bias	Criterion met: Yes A strong association was observed in one study [32]
Dose-response relationship Further evidence of a causal relationship is provided if increasing levels of exposure lead to an increasing risk of disease	Criterion met: Yes A dose-response relationship was observed in one study [34]
Specificity If a particular exposure increases the risk of a certain disease but not the risk of other diseases, this is strong evidence in favour of a cause-effect relationship. However, one-to-one relationships between exposure and disease are rare, and lack of specificity should not be used to say that a relationship is causal	Criterion met: Yes Low birth weight and preterm birth is associated with hip arthroplasty for OA [32] and hip osteophytes [34] but not knee arthroplasty for OA [32] or knee osteophytes [34]
Reversibility When the removal of a possible risk factor results in a reduced risk of disease, the likelihood that this association is causal is increased. Ideally, this should be assessed by conducting a randomized intervention trial. For many exposures or diseases, such randomised trials are not possible in practice	Criterion met: Not applicable for this condition
Coherence The suggested cause-effect relationship should essentially be consistent with the natural history and biology of the disease	Criterion met: No
Analogy The causal relationship will be further supported if there are similarities with other (well-established) cause-effect relationships	Criterion met: Yes Reduced bone mineral density [45] and bone mineral content [10] are found in preterm infants, even when age is corrected for term. Radiological changes, including characteristics of rickets, are identifiable in 23% of infants weighing < 1500 g [58]

### Modelling the economic burden

Table 4 presents national trends in LBW and preterm births in Australia from 2009 to 2014. The proportion of LBW births and preterm births has remained steady over this period (representing 6.4% and 8.0% of all live births in 2014, respectively), although absolute numbers have increased over time in line with an increasing number of births nationwide. A total of 34,321 primary THA procedures were performed in Australia in 2015 and, of these, 30,477 (88.8%) were performed for hip OA [31]. The proportion of study participants undergoing THA who were born with LBW or preterm was 17.3% and 19.0%, respectively [32]. Based on these data [32], 5273 THA procedures performed for OA were estimated as attributable to LBW, while 5791 THA procedures for OA were estimated as attributable to preterm birth. This equates to a total annual cost for THA of AUD $132,150,222 and AUD $145,136,082 for the LBW and preterm populations, respectively.

**Table 4:** Trends in preterm births and low birth weight in Australia

Year	Number of births	Low birth weight births^a^ (%^b^)	Pre-term births^c^ (%^b^)
2009	296,791	18,347 (6.2)	22,645 (7.6)
2010	297,357	18,522 (6.2)	22,952 (7.7)
2011	299,588	18,829 (6.3)	23,282 (7.8)
2012	309,861	19,243 (6.2)	24,671 (8.0)
2013	307,277	19,597 (6.4)	24,582 (8.0)
2014	310,330	19,833 (6.4)	24,826 (8.0)

Data obtained from the Australian Institute of Health and Welfare [19, 29, 30, 59]

^a^ Babies with a weight at birth < 2500 g

^b^ Proportion of all live births for the specified year

^c^ Babies born at 20–36 weeks gestation

## Discussion

In this study, we examined the available evidence for an association between LBW and preterm birth, and the evidence for potential causation based on the Bradford Hill criteria [28]. Considering available data from two high- to fair-quality cohort studies and plausible causation evidence [28], there is a strong indication to support the hypothesis that both LBW and preterm birth are risk factors for hip OA. This has the potential for high healthcare burden since the estimated cost to the Australian health system for THA attributable to LBW and preterm birth would exceed AUD$132 million and AUD$145 million, respectively, based on current estimates. However, the full healthcare costs are likely to be significantly higher, given the additional costs of non-surgical management for less severe hip OA including pain medications and physiotherapy.

Two cohort studies with high to moderate quality and one low-quality cross-sectional study showed that LBW and preterm birth are associated with either hip deformation [36], hip OA [34], or THA for hip OA [32]. Data from the Australian Diabetes, Obesity and Lifestyle Study showed that people with LBW and preterm birth were at higher risk of THA compared with those with normal weight and full-term births, respectively [32]. Similarly, data from the Hertfordshire Cohort Study showed that individuals with lower birth weight were more likely to have hip osteophytes in adulthood; however, there was no relationship between LBW and clinical hip OA as defined by ACR criteria [34]. The ACR criteria specify that hip pain must be present together with radiographic changes to support a diagnosis of hip OA [38]. This diagnostic approach accounts for the known discordance between radiographic findings and symptoms [39]. For example, approximately 85% of people with structural changes indicative of hip OA do not experience frequent hip pain [40]. In another study, 20.1% of individuals with self-reported hip pain had features of clinical hip OA [41]. Thus, making a comparison between radiographic and clinical definitions of hip OA is problematic.

The association between LBW or preterm birth and hip bone abnormality has not always been clear. There was conflicting evidence regarding LBW or preterm birth and hip bone abnormality in three low-quality studies. One cross-sectional study reported that LBW and preterm birth were associated with hip deformity [36]. In contrast, the other cross-sectional study and the only case-control study reported that there was no relationship between LBW or preterm birth, and immature hip and DDH, respectively [35, 37]. There might be several reasons for these discordant findings. For instance, the study that showed LBW and preterm birth were associated with hip deformity measured hip deformity when the children were 3–4 years old [36], while the other study performed ultrasonography to measure hip angle predictive of immature hip predictive of DDH at the gestational age of 40 weeks, regardless of the participant’s actual birth week [37]. It has been shown that a number of infants who progress to hip dysplasia have unstable hips at infancy [42], and hence in most cases dysplasia is diagnosed during late childhood [43]. Furthermore, there is no agreement as to what constitutes an ‘abnormal’ hip [44]. In the case-control study, DDH was self-reported and therefore may have captured only severe cases [35] and failed to show an association.

Examination of Bradford Hill criteria provided further supportive evidence for a causal relationship between LBW and preterm birth and hip OA. Results from the cohort studies supported a temporal relationship between LBW or preterm birth and hip arthroplasty due to hip OA [32], and between LBW and hip osteophytes [34]. There is also some evidence for a “dose-response relationship” between LBW and severity of hip OA, described in one recent cohort study as the lower the birth weight, the higher the likelihood of having osteophytes [34]. Consistent findings were observed using a different spectrum of hip pathology/hip OA including dysplasia [36], osteophytes [34], and arthroplasty [32] of the hip. There was some evidence of specificity as lower birth weight was associated with hip osteophytes but not osteophytes in other joints [34]. In addition, there is evidence of analogy. For example, reduced bone mineral density [45] and bone mineral content [10] are found in preterm infants, even when age is corrected for term. Further work is needed to clarify the role of LBW and preterm birth in the development of hip OA.

The aetiology of hip OA is multifactorial. Both congenital and developmental diseases of the hip, such as mild acetabular dysplasia, may increase the risk of developing of hip OA in adulthood [5, 46]. Preterm babies are born with an incomplete acetabulum at birth [14]. These infants often develop a postural deformation of the legs which persists until early childhood [36] which may be due to an underdeveloped or shallow, upwardly sloping acetabulum [7], decreased joint surface area [47], or lax ligaments holding the ball in place [36]. These factors may influence the structural development of the hip joint, resulting in an abnormal hip joint shape. The important role of hip bone shape and geometry in the aetiology of hip OA has been established [5]. Premature and LBW babies represent a uniquely vulnerable population in which bone growth and mineral acquisition are critical with regards to bone turnover [48]. A case-control study found reduced peak bone mass at the femoral neck in very low birth weight babies [13]. There is emerging evidence that preterm birth and very low birth weight results in a decrease in bone formation and an increase in bone resorption [9, 48] that reduces osteoclast apoptosis [49] and increases cartilage degeneration [50], which may be another potential pathway for the development of hip OA. Furthermore, there might be other mediating factors, i.e. catch-up growth during infancy, high levels of physical activity during puberty, and childhood obesity which, in conjunction with low birth weight and preterm birth, might contribute to hip OA. However, these speculations should be interpreted with caution and more studies are needed to support this hypothesis. If proven to contribute to the development of hip OA, modifying hip position through postural support [15, 16] and perhaps the use of double nappies [51] may be beneficial for these babies. Similarly, it may be that swaddling that forces the hips into extension and adduction, which is a common practice in some Middle East countries and in the US [52], and is having a resurgence in English-speaking countries [53], may predispose to dysplasia and should be discouraged in these babies. Furthermore, these babies should be targeted for hip dysplasia screening even in the absence of overt hip changes; they could also be identified as being at increased risk of hip OA and be considered for preventive strategies as evidence for this emerges. Given the lack of modifiable risk factors for hip OA and with the increasing number of LBW and preterm births, this has the potential to have a major impact on reducing the future burden of hip OA.

Data from the landmark Global Burden of Disease study have shown that the prevalence of hip OA is highest among high-income countries [54] and prevalence is expected to increase with gains in life expectancy. No conventional factors, such as age, body mass index (BMI), or physical activity, fully explain the pathogenesis of the disease [5]. The burden of LBW and preterm birth is also substantial and is increasing internationally [20] with increases in maternal age [30]. In the year 2000 in the US, 55% of all LBW infants were born to women aged 45 years or over [21]. The average age of mothers in Australia has risen from 29.7 years in 2004 to 30.2 years in 2014, and the proportion of mothers aged 35 years and over has increased from 20% in 2004 to 22% in 2014 [30]. This has key health system implications as the age of mothers continues to rise. This study highlights the need to identify babies at risk of developing hip OA, with early assessment of hip joint development among preterm babies and those of LBW and ongoing monitoring of at-risk individuals in childhood and adolescence.

In this study, we performed a systematic literature search with a comprehensive risk of bias assessment. A major limitation of this review is the lack of available studies. However, it is important to note that research into the development and epidemiology of hip OA is generally limited, despite the high burden of disease. One of the included studies used THA for hip OA as a surrogate marker of hip OA and probably has underestimated the association between LBW/preterm birth and hip OA (given that not all individuals with moderate or severe hip OA will undergo hip arthroplasty). However, the study was performed in Australia where there is a publicly funded universal health system (Medicare) and people without private health insurance have access to arthroplasty surgery under this system. The evidence of LBW and preterm birth being risk factors for abnormal hip bone shape, and hip OA was established by applying the Bradford Hill criteria of causation in two high- to fair-quality cohort studies and a few poor-quality case-control and cross-sectional studies. Thus, it is clear that further research is required to determine the influence of LBW and preterm birth in the pathological process of hip OA development. LBW could be due to prematurity (59% to 70% of low birth weight babies) [55, 56], intrauterine growth restriction, or both. In this review, we included either separate or combined preterm birth or LBW data based on how they were examined in the primary study. It is therefore not possible to draw conclusions as to how low birth weight alone, low birth weight along with preterm birth, or preterm birth alone contributes to the pathophysiology of hip OA.

The LBW and preterm populations were treated separately for the economic evaluation, although there will undoubtedly be some overlap between these groups. The THA costs are based on average costs for an episode of care and cannot account for individual differences in arthroplasty costs. Finally, our analysis does not include the indirect or out-of-pocket costs of THA.

## Conclusion

Despite the lack of high-quality studies in this area, our findings suggest that LBW and preterm birth are potential risk factors for hip bone shape abnormalities and hip OA requiring THA in adulthood. Based on our calculations, this could have substantial financial implications for healthcare systems. Given the current lack of effective treatment and preventive strategies for hip OA, this is an area where further research is needed to reduce the burden of hip OA. For example, the individuals born preterm or with LBW may be identified as an “at-risk group” for future end-stage hip OA; this will enable targeted monitoring and early interventions that could potentially reduce the population burden of THA in later life.

## Additional file

Additional file 1: **Table S1.** Risk of bias assessment of cohort and cross-sectional studies. **Table S2.** Risk of bias assessment of case-control studies. (DOCX 28 kb)

## Abbreviations

ACR: American College of Rheumatology
DDH: Developmental dysplasia of the hip
K-L: Kellgren and Lawrence
LBW: Low birth weight
MRI: Magnetic resonance imaging
NHLBI: National Heart Lung and Blood Institute
OA: Osteoarthritis
PRISMA: Preferred Reporting Items for Systematic Review and Meta-Analysis
THA: Total hip arthroplasty
