# Supplementary material for: Could low birth weight and preterm birth be associated with significant burden of hip osteoarthritis? A systematic review
Source: Arthritis Res Ther. 2018 Jun 8;20:121. doi: 10.1186/s13075-018-1627-7 (PMC5994049; doi:10.1186/s13075-018-1627-7)
Supplement: Supplementary file 1 — Table S1. Risk of bias assessment of cohort and cross-sectional studies. Table S2. Risk of bias assessment of case-control studies. (DOCX 28 kb) [file 13075_2018_1627_MOESM1_ESM.docx]

**Table S1: Risk of bias assessment of cohort and cross-sectional studies**

| Study | Criteria 1 | Criteria 2 | Criteria 3 | Criteria 4 | Criteria 5 | Criteria 6 | Criteria 7 | Criteria 8 | Criteria 9 | Criteria 10 | Criteria 11 | Criteria 12 | Criteria 13 | Criteria 14 | Overall quality |
| --- | --- | --- | --- | --- | --- | --- | --- | --- | --- | --- | --- | --- | --- | --- | --- |
| COHORT | | | | | | | | | | | | | | |  |
| Hussain 2015 (Australia)[31] | yes | yes | yes | yes | yes | yes | yes | no | yes | NA | yes | yes | yes | yes | High |
| Clynes 2014  (United Kingdom)[33] | yes | yes | yes | yes | no | yes | yes | yes | yes | NA | yes | NR | no | yes | Fair |
| CROSS-SECTIONAL | | | | | | | | | | | | | | |  |
| Davis 1993  (United Kingdom)[35] | yes | yes | NR | no | no | no | no | NA | no | NA | no | NR | NA | no | Low |
| Orak 2015  (Turkey)[36] | yes | yes | yes | yes | yes | no | no | yes | yes | NA | yes | NR | NA | no | Low |

NA = not applicable NR= not reported

Items included on the risk of bias tool:

1. Was the research question or objective in this paper clearly stated?
2. Was the study population clearly specified and defined?
3. Was the participation rate of eligible persons at least 50%?
4. Were all the subjects selected or recruited from the same or similar populations (including the same time period)? Were inclusion and exclusion criteria for being in the study pre-specified and applied uniformly to all participants?
5. Was a sample size justification, power description, or variance and effect estimates provided?
6. For the analyses in this paper, were the exposure(s) of interest measured prior to the outcome(s) being measured? (For cross-sectional analyses, the answer to Question 6 should be "no.")
7. Was the timeframe sufficient so that one could reasonably expect to see an association between exposure and outcome if it existed? (For cross-sectional analyses, the answer to Question 6 should be "no.")
8. For exposures that can vary in amount or level, did the study examine different levels of the exposure as related to the outcome (e.g., categories of exposure, or exposure measured as continuous variable)? (if binary NA)
9. Were the exposure measures (independent variables) clearly defined, valid, reliable, and implemented consistently across all study participants?
10. Was the exposure(s) assessed more than once over time?
11. Were the outcome measures (dependent variables) clearly defined, valid, reliable, and implemented consistently across all study participants?
12. Were the outcome assessors blinded to the exposure status of participants?
13. Was loss to follow-up after baseline 20% or less?
14. Were key potential confounding variables measured and adjusted statistically for their impact on the relationship between exposure(s) and outcome(s)?

**Table S2: Risk of bias assessment of case-control studies**

| Study | Criteria 1 | Criteria 2 | Criteria 3 | Criteria 4 | Criteria 5 | Criteria 6 | Criteria 7 | Criteria 8 | Criteria 9 | Criteria 10 | Criteria 11 | Criteria 12 | Overall quality |
| --- | --- | --- | --- | --- | --- | --- | --- | --- | --- | --- | --- | --- | --- |
| **Case-control studies** | | | | | | | | | | | | |  |
| Chan 1997 (Australia)[34] | yes | yes | yes | yes | no | yes | NA | yes | yes | no | NR | yes | Low |

Items included on the risk of bias tool:

1. Was the research question or objective in this paper clearly stated and appropriate?

2. Was the study population clearly specified and defined?

3. Did the authors include a sample size justification?

4. Were controls selected or recruited from the same or similar population that gave rise to the cases (including the same timeframe)?

5. Were the definitions, inclusion and exclusion criteria, algorithms or processes used to identify or select cases and controls valid, reliable, and implemented consistently across all study participants?

6. Were the cases clearly defined and differentiated from controls?

7. If less than 100 percent of eligible cases and/or controls were selected for the study, were the cases and/or controls randomly selected from those eligible?

8. Was there use of concurrent controls?

9. Were the investigators able to confirm that the exposure/risk occurred prior to the development of the condition or event that defined a participant as a case?

10. Were the measures of exposure/risk clearly defined, valid, reliable, and implemented consistently (including the same time period) across all study participants?

11. Were the assessors of exposure/risk blinded to the case or control status of participants?

12. Were key potential confounding variables measured and adjusted statistically in the analyses? If matching was used, did the investigators account for matching during study analysis?
